# A meta-analysis approach for assessing the diversity and specificity of belowground root and microbial volatiles

**DOI:** 10.3389/fpls.2015.00707

**Published:** 2015-09-14

**Authors:** Denis Schenkel, Marie C. Lemfack, Birgit Piechulla, Richard Splivallo

**Affiliations:** ^1^Institute for Molecular Biosciences, Goethe University FrankfurtFrankfurt, Germany; ^2^Integrative Fungal Research ClusterFrankfurt, Germany; ^3^Institute for Biological Sciences, University of RostockRostock, Germany

**Keywords:** microbes, fungi, bacteria, volatiles, diversity, rhizosphere, mycorrhizas, roots

## Abstract

Volatile organic compounds are secondary metabolites emitted by all organisms, especially by plants and microbes. Their role as aboveground signals has been established for decades. Recent evidence suggests that they might have a non-negligible role belowground and might be involved in root–root and root–microbial/pest interactions. Our aim here was to make a comprehensive review of belowground volatile diversity using a meta-analysis approach. At first we synthesized current literature knowledge on plant root volatiles and classified them in terms of chemical diversity. In a second step, relying on the mVOC database of microbial volatiles, we classified volatiles based on their emitters (bacteria vs. fungi) and their specific ecological niche (i.e., rhizosphere, soil). Our results highlight similarities and differences among root and microbial volatiles and also suggest that some might be niche specific. We further explored the possibility that volatiles might be involved in intra- and inter-specific root–root communication and discuss the ecological implications of such scenario. Overall this work synthesizes current knowledge on the belowground volatilome and the potential signaling role of its constituents. It also highlights that the total diversity of belowground volatiles might be orders of magnitude larger that the few hundreds of compounds described to date.

## Introduction

Secondary metabolites are small molecules that are produced by all living organisms. Unlike primary metabolites which are directly involved in regular growth and development, secondary metabolites might be produced only at specific developmental stages or under certain circumstances; hence they might provide a functional readout of cellular state (Patti et al., 2012). Tens of 1000s of secondary metabolites derived from plants and microbes are known to humans as drugs, food additives or flavors, and fragrances; yet, their ecological functions remain poorly understood.

Secondary metabolites indeed play a central role in inter-organismic interactions. In numerous cases volatile and non-volatile secondary metabolites have been implicated in defense and communication among organisms. Recently, volatiles have attracted sustained attention, especially in belowground communication, due to their ability to travel further distances than non-volatile metabolites (Rasmann et al., 2005; Wenke et al., 2010; Peñuelas et al., 2014). Because of their potent biological activities on plants, the use of volatiles in agriculture have been suggested as a possible alternative to pesticides (Bitas et al., 2013; Kanchiswamy et al., 2015). A search through literature and databases allows estimating the known structural diversity of volatiles derived from plant flowers – about 1700 volatiles from 991 species (Knudsen et al., 2006; Dunkel et al., 2009) – and from microbes, including fungi and bacteria – 1093 volatiles from 491 microbes at the time of this study (Lemfack et al., 2014). Yet considering that 10^7^–10^9^ bacterial species (Schloss and Handelsman, 2004), 1.5 million fungal species (Hawksworth, 2001) and 2,98,000 of plant species (Mora et al., 2011) might exist on earth, the number of volatiles will increase as new species are being characterized and discovered.

In the past 5 years, the ecological role of volatiles in above- and belowground interactions among plants, fungi, bacteria, and insects has been addressed in a series of comprehensive reviews (Wenke et al., 2010, 2012; Bailly and Weisskopf, 2012; Effmert et al., 2012; Davis et al., 2013; Farag et al., 2013; Audrain et al., 2015; Kanchiswamy et al., 2015; Schmidt et al., 2015). The latest of these reviews (Kanchiswamy et al., 2015) covered literature up to the beginning of 2015. Most recently a further example of belowground volatile based communication has been brought to light for plants and the ectomycorrhizal fungus *Laccaria bicolor* (Ditengou et al., 2015). Some volatile sesquiterpenoids emitted by the latter fungus were shown to induce root branching in poplar, a host plant which can enter into symbiotic interactions with the fungus, but also in *Arabidopsis*, a non-host plant unable of symbiosis with *Laccaria*. Remarkably not all fungal sesquiterpenoids induced root branching: the volatile (–)-thujopsene was implicated in the root morphological change but β-caryophyllene, another sesquiterpenoid also emitted by maize roots (Rasmann et al., 2005), had no effect on branching. These observations raise questions about the specificity of belowground signals as well as the ability of the target organisms to perceive and react to volatiles.

Soil is actually a highly colonized inhomogeneous substrate. Non-homogeneity is not only reflected in terms of structure and porosity but also in terms of nutritional differences (Schoenholtz et al., 2000). Besides, organisms present in the soil might also provide specific niches for defined microbes, thus exerting a community structuring effect. Belowground community structuring has indeed been observed in numerous cases. A textbook example includes root nodules in legumes which are exclusively colonized by nitrogen fixing rhizobacteria (Gage, 2004). More recent examples are provided by *Arabidopsis’* root endophytic microbial community made of *Proteobacteria*, *Bacteroidetes*, and *Actinobacteria* (Bulgarelli et al., 2012) and by truﬄe’s fruiting bodies which host bacterial communities clearly distinct from those of the surrounding soil (Antony-Babu et al., 2014). This belowground community structuring might explain why some volatiles could act as successful signaling cues within such communities, however evidence that specific volatiles are emitted in defined habitats/niches is currently limited.

The aim of this paper is to quantify the diversity and explore the specificity of belowground volatiles produced by microbes and plant roots. For this purpose we synthesized existing literature on plant root volatiles and relied on the “mVOC database” of microbial volatiles (Lemfack et al., 2014) to address questions such as: how structurally diverse are plant root and microbial volatiles? Which volatiles are common and specific to microbes and plant roots? Is their emission influenced by microbial phylogeny or habitat; and finally do root volatiles serve as signals for neighboring plants? Overall our aim was to shed more light on belowground volatiles diversity and functions by essentially using a quantitative approach to diversity and by integrating information on the phylogeny and the habitat of the emitters.

## Materials and Methods

### Diversity of Plant Root Volatiles

**FIGURE 1:**
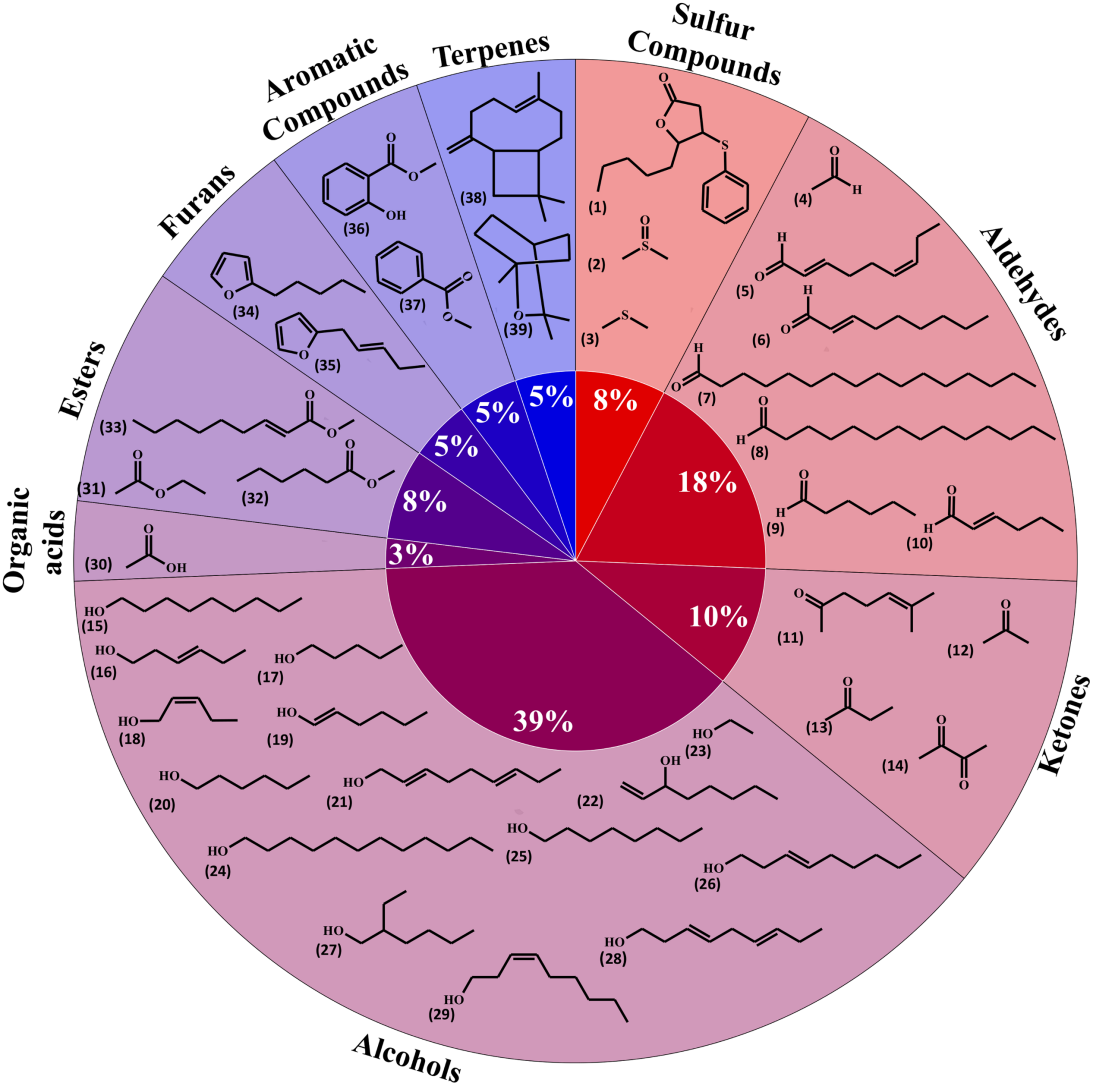
**Diversity of plant root volatiles**. A total of 39 volatiles released from barley, maize, *Arabidopsis* or fava bean roots were classified based on their chemical classes and shown here in a pie chart. 5-Pentyl-4-phenylsulfanyloxolan-2-one (1); dimethyl sulfoxide (2); dimethyl sulfide (3); acetaldehyde (4); (2*E*,6*Z*)-nona-2,6-dienal (5); (*E*)-non-2-enal (6); hexadecanal (7); tetradecanal (8); hexanal (9); (2*E*)-hex-2-enal (10); 6-methylhept-5-en-2-one (11); acetone (12); butan-2-one (13); butane-2,3-dione (14); nonan-1-ol (15); (3*E*)-hex-3-en-1-ol (16); pentan-1-ol (17); (2*Z*)-pent-2-en-1-ol (18); (1*E*)-hex-1-en-1-ol (19); hexan-1-ol (20); nona-2,6-dien-1-ol (21); oct-1-en-3-ol (22); ethanol (23); dodecan-1-ol (24); octan-1-ol (25); (*E*)-non-3-en-1-ol (26); 2-ethylhexan-1-ol (27); nona-3,6-dien-1-ol (28); (*Z*)-non-3-en-1-ol (29); acetic acid (30); ethyl acetate (31); methyl hexanoate (32); methyl (*E*)-non-2-enoate (33); 2-pentylfuran (34); 2-pent-2-enylfuran (35); methyl salicylate (36); methyl-benzoate (37); β-caryophyllene (38); 1,8-cineole (39).

Volatile organic compounds (VOCs) released by plant roots have been investigated in a limited number of species. Here we gathered information relative to volatile diversity in barely – *Hordeum vulgare* – (29 compounds; Gfeller et al., 2013), the model plant *Arabidopsis thaliana* (eight compounds; Steeghs et al., 2004), maize – *Zea mays* (one compound; Rasmann et al., 2005) and the bean *Vicia faba* (one compound; Babikova et al., 2013). Overall these plant roots emitted 39 volatiles, which have been grouped in **Figure 1** based on their biosynthetic origins/chemical classes (i.e., terpenoids, alcohols).

### Diversity of Microbial Volatiles

**FIGURE 2:**
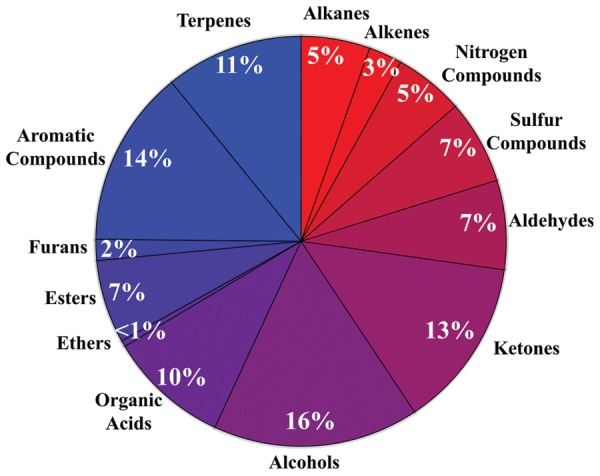
**Diversity of microbial volatiles**. The 1093 volatiles from the mVOC database (Lemfack et al., 2014) grouped in chemical classes are shown as a pie chart.

The diversity of microbial volatiles was investigated using the mVOC database (Lemfack et al., 2014). At the time of this study, the database comprised 1093 volatiles emitted by 135 fungi and 356 bacteria. As for plant roots, volatiles were classified according to chemical classes/biosynthetic origins (**Figure 2**).

### Specificity of Microbial Volatiles Linked to Taxonomy and Ecological Niches

To understand how specific or common volatiles were in microbes, bacteria and fungi of the mVOC database were classified in taxonomical units either at the phylum or class level. Gaining insight into niche specificity was achieved by classifying the microbes of the mVOC database based on their habitat. Because of our focus on belowground interactions, classification was made in five categories: fungi or bacteria living in the rhizosphere, fungi, or bacteria living in the soil (excluding the rhizosphere), and microbes living in any other habitat (i.e. animals, marine habitats, and microorganisms associated to above-ground plant parts). Classification in specific niches/habitats was based on various data sources which will be shortly included in the mVOC database. Because we were interested in habitat/niche specificity, microbes which were ubiquitous to more than one habitat/niche were excluded from the analysis.

### Effect of Neighboring Plant on Root Development

The influence of neighboring plants on root development was investigated by compiling data from 18 publications (Mahall and Callaway, 1992; Gersani et al., 2001; Maina et al., 2002; Day et al., 2003; Falik et al., 2003, 2006; Gruntman and Novoplansky, 2004; O’Brien et al., 2005; Dudley and File, 2007; Murphy and Dudley, 2007, 2009; Semchenko et al., 2007, 2014; Broz et al., 2008; Milla et al., 2009; Fang et al., 2013; Schmid et al., 2013a). In all those works root development (biomass or root length depending on the parameter reported) of a plant subjected to neighboring plants was compared to root development of a plant without neighbors. The effects on roots were classified as “increase, decrease, no effect” based on the statistics reported in the papers. Subject plants were grouped either based on genetic relatedness with the interacting plants (as kin, conspecific but not kin, and foreign species) or as monocots and dicots. Cases in which the kinship of individuals of the same species was unspecified were categorized as conspecific.

## Results

### Diversity of Plant Root and Microbial Volatiles

Whereas publications investigating volatiles emitted by aboveground plant organs abound, only a few papers have been published on root volatiles, most likely due to the technical difficulties in sampling volatiles in soil matrices. Compiling the information from root volatiles emitted by maize (Rasmann et al., 2005), barley (Gfeller et al., 2013), *Arabidopsis thaliana* (Steeghs et al., 2004), and the bean *Vicia faba* (Babikova et al., 2013) revealed an overall diversity of 39 volatiles belonging to nine chemical/biosynthetic groups (**Figure 1**). With 66% of all volatiles, alcohols, aldehydes, and ketones represented the major share of root volatiles. The remaining 44% was composed of minor groups (sulfur compounds, terpenoids, aromatic compounds, furans, esters, and organic acids) each represented by a single or two compounds. By contrast to the scarce information on root volatiles, microbial volatiles have been investigated more thoroughly. An effort to synthesize the large amount of information on microbial volatiles has recently been made through the mVOC database (Lemfack et al., 2014), which also served as the basis of the present study. Here a total of 1093 microbial volatiles from the mVOC database have been grouped according to chemical classes/biosynthetic pathways and the resulting data is presented as a pie chart in **Figure 2**. Even though some volatiles like ketones, esters, sulfur-containing compounds, and furans appeared with a comparable frequency as in plants roots and microorganisms, the microbial volatilome comprised a greater structural complexity of organic acids, aromatic compounds, and terpenes than plant roots, at least considering the currently available data (**Figures 1** and **2**). Five groups of microbial volatiles (terpenes, alcohols, ketones, aromatic compounds, and organic acids) represented each 10% or more of the volatiles, overall accounting for 64% of the total diversity. Aldehydes, sulfur and nitrogen containing compounds, alkanes, alkenes, furans, ester, and ethers represent minor groups accounting together for almost 37% of the total diversity.

### Which Microbes Produce Plant Root Volatiles?

**FIGURE 3:**
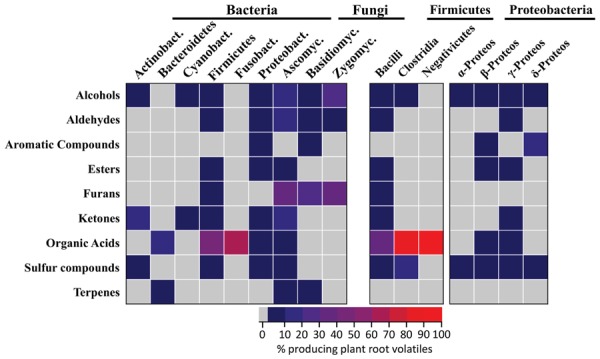
**Which microbes produce plant root volatiles?** Bacteria and fungi producing the same volatiles as plant roots (**Figure 1**) were identified from the mVOC database. The results are displayed for the different phyla or classes of bacteria and fungi as a heatmap where each cells represents the percentage of microbes emitting volatiles of **Figure 1**. Actinobact: *Actinobacteria* (*n* = 64), *Bacteroidetes* (*n* = 44), Cyanobact: *Cyanobacteria* (*n* = 19), *Bacilli* (*n* = 11), *Clostridia* (*n* = 10), *Negativicutes* (*n* = 1), Fusobact: *Fusobacteria* (*n* = 3), α-Proteos: α-*Proteobacteria* (*n* = 25), β-Proteos: β-*Proteobacteria* (*n* = 43), γ-Proteos: γ-*Proteobacteria* (n = 62), δ-Proteos: δ-*Proteobacteria* (*n* = 17), Ascomyc: *Ascomycota* (*n* = 104), Basidiomyc: *Basidiomycota* (*n* = 28), Zygomyc: *Zygomycota* (*n* = 3).

A total of 28 plant root volatiles were also produced by microbes. These volatiles included 11 alcohols (dodecan-1-ol; ethanol; 2-ethyl-1-hexanol; hexan-1-ol; 2-hexen-1-ol; 3-hexen-1-ol; 1-nonanol; 1-octanol; 1-octen-3-ol; pentanol; 2-penten-1-ol), 4 aldehydes (acetaldehyde; hexanal; 2-hexenal; tetradecanal), two aromatic compounds (methyl benzoate; methyl salicylate), two esters (ethyl acetate; methyl hexanoate), one furan (2-pentylfuran), four ketones (acetone; butanone; butanedione; 6-methyl-5-hepten-2-one), one organic acid (acetic acid), two sulfur compounds (dimethyl sulfide; sulfinylbismethane), and one terpene (β-caryophyllene). Our aim was to understand if these volatiles were preferentially produced by specific bacterial or fungal phyla/classes. For this purpose, microbes emitting plant root volatiles were grouped in phyla and in some cases in classes. The heatmap in **Figure 3** represents the percentage of microbes, which are emitters of the plant root volatiles of **Figure 1**.

In terms of volatile groups, 14 fungal and 22 bacterial phyla emitted plant root volatiles at a rather low frequency (<10% as shown from the color scale on the heatmap of **Figure 3**). Alcohols were the most frequent and were emitted by four of the seven bacterial phyla and by all the fungal phyla. Volatiles belonging to remaining groups were similarly emitted at a low frequency by 50% of all phyla (fungal and bacterial). Interestingly all volatile groups occurred in at least one fungal and one bacterial phylum. Furans were produced by a fair percentage of fungi belonging to the three fungal phyla considered here whereas it was emitted at low frequency within a single bacterial phylum (*Firmicutes*, specifically the *Bacilli* class).

Considering the data in terms of phyla highlighted that members of the *Firmicutes* and *Proteobacteria* bacterial phyla and *Ascomycetes* fungi emitted volatiles belonging to most of the chemical groups. Zooming into bacterial classes revealed that among the *Firmicutes*, *Bacilli* were the most frequent emitters of plant root volatiles while among the *Proteobacteria*, β-, and γ-*Proteobacteria* were the most frequent emitters. Interestingly, acetic acid (the only molecule in the category “organic acid”) was produced by about 50% of all *Bacilli* and an even higher percentage of *Clostridia* (the highest percentage with Negativicutes reflects the fact that this class has a single representative).

Overall these results highlight that numerous microbes are capable of emitting the same volatiles as plant roots. They also suggest that some phyla might be better than others at producing these volatiles. Bacteria belonging to the *Firmicutes* (*Bacilli*), to the *Proteobacteria* (β- and γ-*Proteobacteria*) and *Ascomycetes* fungi specifically stand out for their ability to produce a large variety of plant root volatiles.

### Common and Specific Volatiles to Plant Roots, Bacteria, and Fungi

The microbial volatiles of the mVOC database and the plant root volatiles of **Figure 1** have been presented according to the potential origin/habitat of their emitters. These origins have been regrouped here in five categories as plant roots (39 volatiles), rhizosphere fungi (261 volatiles), rhizosphere bacteria (209 volatiles), soil fungi (187 volatiles), soil bacteria (483 volatiles). The data is presented as a Venn diagram highlighting the number of specific and common volatiles among groups (**Figure 3**).

A total of 853 volatiles were emitted by plant roots and belowground microbes. Considering the five groups defined here, all groups shared eight volatiles; however, the majority of volatiles were unique to distinct origins/habitats. For example, of the 39 volatiles produced by plant roots (**Figure 1**), 12 (or 31%) were solely produced by roots and not by any other microbes. Depending on their habitat fungi produced 145 (rhizosphere) and 96 (soil) unique volatiles not shared by any other groups; by contrast soil and rhizosphere fungi had 61 volatiles in common. The same argument can be made for bacteria, which produced 76 (rhizosphere) and 297 (soil) unique volatiles, and shared 126 of them.

Overall this data exemplifies the specificity but also the extent of the overlap in volatile signals emitted by plant roots and microbes. It highlights the existence of a core volatilome for bacteria and plant roots but also the fact that a high proportion of volatiles are specific to organisms in defined habitats.

### Are Microbial Volatiles Niche Specific?

**FIGURE 4:**
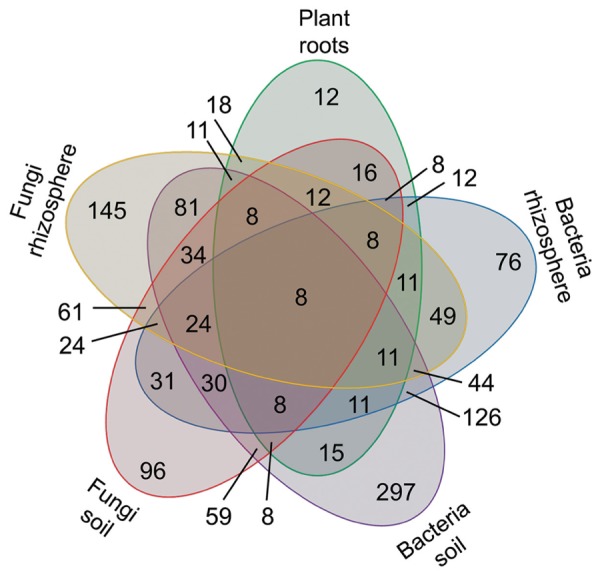
**Number of volatiles specific and common to plant roots, bacteria, and fungi**. A total of 853 volatiles emitted by plant roots (**Figure 1**) and belowground microbes were grouped based on their source/habitat in the five categories and are displayed in a Venn diagram. The numbers of volatiles specific or shared among categories are displayed here.

Fungi and bacteria from the mVOC database were regrouped according to their lifestyle/habitat. Similarly to **Figure 4** three categories were considered in relation to possible interactions with plants: organisms typically found in the soil (S), microbes associated with the rhizosphere (R) and organisms which did not fall in those two categories (N) (i.e., either associated to above plant organs or with animals). Only volatiles occurring in at least 10 microbes are shown here. Values in the heatmap represent the percentage of microbes emitting a specific volatile in each category.

In terms of chemical classes/groups, numerous terpenes, aromatic compounds, nitrogen, and sulfur containing compounds, alkanes and alkenes were predominantly produced by bacteria compared to fungi. Some volatiles such as nitrogen containing compounds were actually almost exclusively produced by bacteria. By contrast no volatiles were exclusively produced by fungi. In most cases habitat specificity (i.e., soil, rhizosphere) seemed to have little influence on volatiles patterns. Volatiles belonging to a few groups were, however, predominantly produced by rhizosphere (R) organisms (in opposite to soil (S) and “other” (N) organisms). This was the case for example in fungi for alcohols, sulfur compounds, some aromatic compounds (i.e., 2-phenylethanol) and some ketones (i.e., octan-3-one). Similarly in bacteria nitrogen containing compounds production seemed slightly higher in rhizosphere organisms.

**FIGURE 5:**
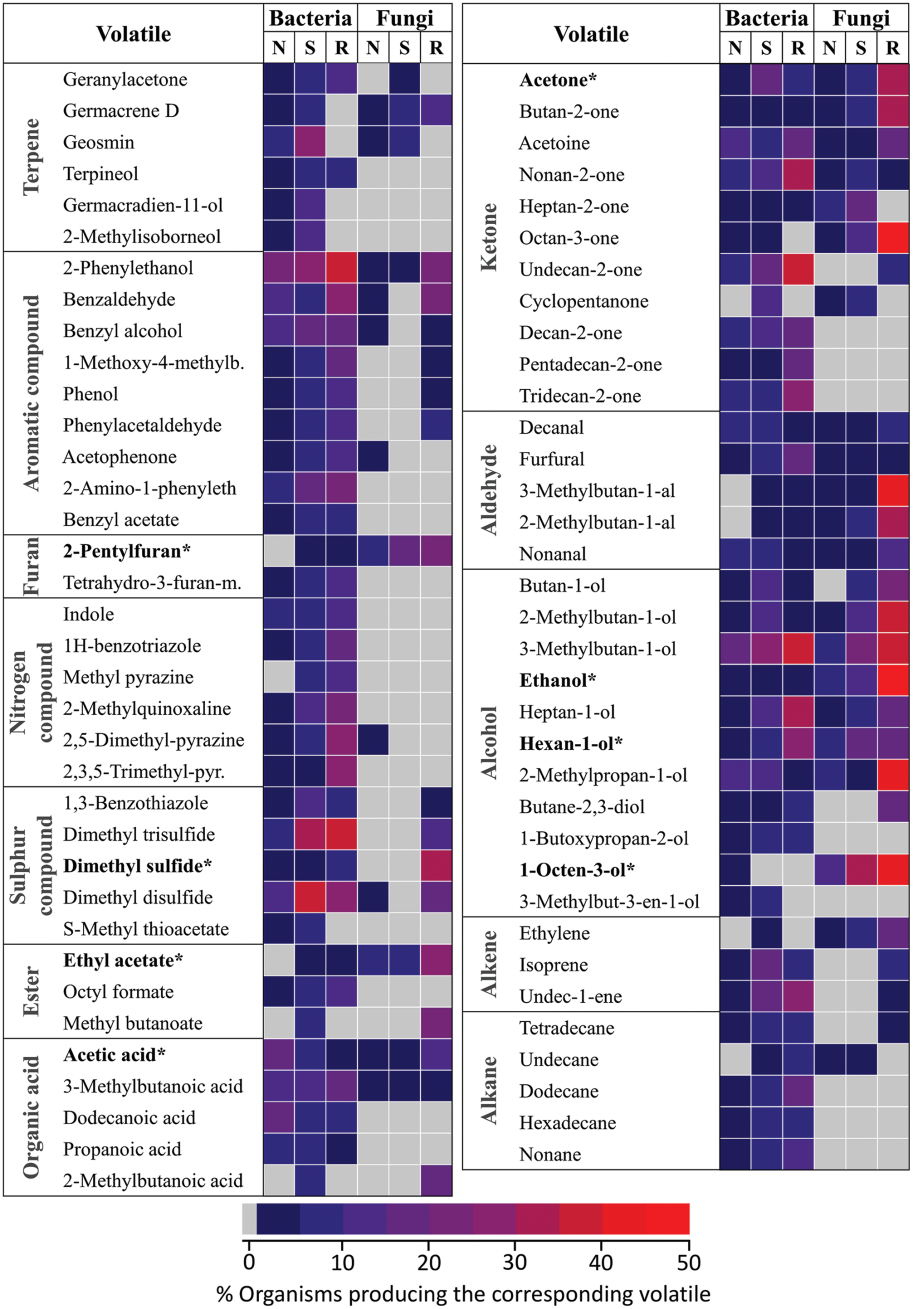
**Niche specificity of bacterial and fungal volatiles**. Volatiles emitted by at least 10 different organisms from the mVOC database are shown. Microbes were classified based on their lifestyle/habitat as “soil” (120 bacteria, 36 fungi), “rhizosphere” (rhizo: 43 bacteria, 26 fungi) and “other” (157 bacteria, 64 fungi). The color code shows the frequency of each volatile in each group. ^∗^These volatiles are also emitted by plant roots.

Plant root volatiles shown in bold were marked with an asterisk in **Figure 5**. With the exception of 1-octen-3-ol, most of these volatiles were emitted by microbes in most/all categories. Nevertheless it is noteworthy that six of the eight plant root volatiles shown here [2-pentylfuran; dimethyl sulfide (syn. (methylsulfanyl)methane]; ethyl acetate; acetone; ethanol; 1-octen-3-ol) were emitted by a comparatively higher percentage of rhizospheric fungi compared to fungi and bacteria colonizing different habitats.

These results demonstrate marked differences in terms of volatile production patterns among bacteria and fungi. This suggests that bacteria might be capable of synthesizing structurally more diverse volatiles than fungi. They also indicate that microbes belonging to specific niches/habitats, especially to the rhizosphere, might preferentially produce volatile signals, including many of the volatiles also emitted by plant roots.

### Could Root Volatiles be Perceived by Neighboring Plants?

**FIGURE 6:**
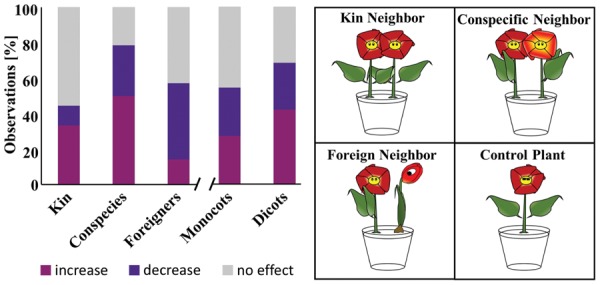
**Influence of a neighboring plant on root development**. Data presented in a bar chart summarizes the observations of several studies which compared root development of a plant with and without neighbor. The cartoon on the right illustrates the interactions considered here. “Increase, decrease, no effect” refer to the root biomass of a plant with neighbor compared to the one without. Kin: 20 observations in which interacting plants are kins. Conspecies: 27 observations where tested plants are from the same species (but not kins). Foreign: 45 observations where interacting plants belong to different species. Monocots: 28 observations involving monocotyledonous plants. Dicot: 64 observations involving dicotyledonous plants.

There is a mounting body of evidence that neighboring plants can communicate with each other through their roots (Dudley and File, 2007; Bhatt et al., 2011; Fang et al., 2013; Schmid et al., 2013b). Obvious signals for such communication might be volatile molecules. Additionally, volatile emission patterns of aboveground plant organs were shown to be dependent on genetic relatedness. For the sake of clarity, kin plants by definition share the same parents/ancestors, as opposed to conspecific plants which, besides belonging to one species, do not have common parents/ancestors. Recently volatile profiles of kins were shown to be more similar to each other than those of plants without kinship (conspecific plants) (Karban et al., 2014). This led us to question whether plant roots react differently to neighboring plants based on their genetic relatedness (i.e., kins, conspecific but not kins, or foreign (different species) – see cartoon of **Figure 6**). Furthermore we also questioned if differences existed among monocots and dicots.

To answer these questions we gathered publications, which compared root development of one plant with a neighbor to a single plant without neighbor. A total of 30 observations from 18 publications were taken into account and their outcomes have been synthesized in **Figure 6**.

Comparing kins to conspecifics revealed that the roots of more than 50% of kins were unaffected by their neighbors compared to only 21% for conspecifics. When an effect was observed, this predominantly corresponded to an increased root biomass for both categories. The opposite was true for plants subjected to a foreign neighbor. These predominantly (43% of all observations) reacted to the neighbor by decreasing or shortening their roots. Patterns were less obvious with monocots and dicots. Indeed the number of cases in which roots were either affected (increase or decrease in biomass/root length) or unaffected were comparable.

Overall these results highlight that among kin, foreign and conspecific plants, roots of kins are the less likely to be influenced by a neighbor. They also exemplify that plants sharing the same genotype (kins and conspecifics) might predominantly react to each other by increasing their root biomass/root length, while plants with a foreign neighbor might commonly decrease their root biomass.

## Discussion

During the past decade VOCs have gained recognition as essential signals in inter-organismic interactions. Especially belowground volatiles might convey information among plant roots, microbes, and insects. The diversity of volatiles and effects on their target organisms have been recently synthesized in a series of comprehensive reviews (Wenke et al., 2010, 2012; Bailly and Weisskopf, 2012; Effmert et al., 2012; Davis et al., 2013; Kanchiswamy et al., 2015). Our aim here was to bring this synthesis one step further by using a quantitative meta-analysis approach and integrating data about phylogeny and potential habitat of the emitters.

### Diversity of Belowground Volatiles

Adding up volatiles emitted by plant roots to volatiles emitted by soil/root microbes brings the total diversity of belowground volatiles to 853. Considering the scarce information on plant root volatiles (only a few existing publications), and the huge unexplored diversity of soil microbes, the overall diversity of belowground volatiles might be orders of magnitudes higher than the few 100s of compounds described to date.

A note of caution should nevertheless be used when estimating diversity from literature data, since most studies describing volatiles from microbes or plant roots have been conducted under laboratory (and sometimes axenic) conditions. Indeed it is well known that media composition, culture conditions or interacting organisms might influence secondary metabolism (Blom et al., 2011; Brakhage and Schroeckh, 2011). Hence if the presence of one volatile in the mVOC database reflects the ability of specific organisms to produce that volatile, its absence does not exclude that it might be produced under natural conditions. The reverse is certainly also true. Overall estimating the total diversity of belowground volatiles will require isolating and characterizing more microbes/plant roots but also analyzing full soil communities under both laboratory and natural conditions. It should be highlighted that profiling volatiles from soil is much more complicated than from any other system. Indeed soil is a highly complex matrix which requires the most advanced instrumentation in terms of resolution and sensitivity (i.e., high resolution MS or proton transfer MS) as well as powerful data processing for harnessing the complexity of its volatilome (Peñuelas et al., 2014; Mancuso et al., 2015).

### Differences and Similarities in Volatile Profiles of Roots, Bacteria, and Fungi

Our analysis highlighted similarities and differences among plant root and belowground microbial volatiles. In terms of similarities, numerous microbes were capable of emitting the same volatiles as plant roots; however, bacteria belonging to the *Firmicutes* (*Bacilli*), to the *Proteobacteria* (β- and γ-*Proteobacteria*) and *Ascomycete* fungi especially distinguished themselves in this regard. Interestingly *Firmicutes* and β- and γ-*Proteobacteria* tend to be dominant root endophytes in rice and sugarcane (Fischer et al., 2011; Sessitsch et al., 2012). Ascomycete fungi also include numerous members which live in close association with plant roots (i.e., truﬄes forming ectomycorrhizas; Martin et al., 2010). It is therefore tempting to speculate that resemblance in terms of volatile profiles might translate into closer associations between microbes and plant roots. Testing this hypothesis will require characterizing the volatile profiles of numerous plant roots and microbial strains under natural conditions.

### How Specific are Belowground Volatile Signals?

Determining how specific volatile signals might be in terms of interactions requires understanding the nature of the interaction and also the habitat in which it takes place. In terms of molecules, terpenoids are not only important volatiles for floral scent (Knudsen et al., 1993, 2006; Pichersky and Gershenzon, 2002), but as illustrated by β-caryophyllene in maize, they might serve as an alarm signal upon attack by root pests (Rasmann et al., 2005). This volatile is also emitted by a bacterium belonging to the *Bacteroidetes* phylum and by some fungi (**Figure 3**; Lemfack et al., 2014). It has additionally been reported from the fungus *Fusarium oxysporum* colonized by ectosymbiotic bacteria, and it is responsible of the growth promoting effect observed in lettuce colonized by the latter fungus (Minerdi et al., 2011). This example illustrates that one volatile might be produced by numerous organisms to a different end. Another terpenoid, geosmin, which is produced by numerous microbes (Lemfack et al., 2014) was also recently reported from beet roots (*Beta vulgaris* sp. *vulgaris*) (Freidig and Goldman, 2014). We had originally not included this compound in our list of plant root volatiles because they were suspicions that it might not have been produced by beet root itself but by microbes colonizing beet roots tissues; however, the data presented by Freidig and Goldman (2014) suggests that this might be otherwise. This highlights that characterizing the volatile profiles of existing plant roots might greatly increase the diversity of plant root volatiles.

Sulfur containing volatiles are also important signals in plant–microbe interactions. Indeed it has recently been demonstrated that dimethyl disulfide produced by Bacillus bacteria naturally colonizing tobacco roots promoted plant growth by enhancing sulfur assimilation (Meldau et al., 2013). Our data highlights that this volatile is predominantly produced by bacteria (essentially soil bacteria) and to a lesser extent by fungi (**Figure 5**). A tempting interpretation might be that numerous soil bacteria might use this volatile for plant growth promotion. Other bacterial volatiles might also serve this purpose, indeed 2,3-butanediol promotes plant growth in Arabidopsis (Ryu et al., 2003). Nevertheless the overall effect of microbial volatiles on plant growth depends on the total volatile blend and cultural conditions of the microbes (Blom et al., 2011; Peñuelas et al., 2014). Therefore understanding the specificity of a signal also requires characterizing the context in which it is emitted as well as the bioactivity of the total volatile blend.

Eight carbon containing volatiles are characteristic of fungi, and its major representative, 1-octen-3-ol is indeed responsible of the typical fungal smell perceived by humans (Wnuk et al., 1983; Mosandl et al., 1986). Our data indicates that 1-octen-3-ol and octan-3-one are predominantly produced by rhizospheric fungi (**Figure 5**). Since numerous of these fungi live in symbiotic association with plant roots (i.e., truﬄes), eight carbon-containing volatiles might serve as symbiotic signals to a potential host plant. In terms of biological activity high concentrations of these volatiles have been shown to inhibit seed germination and seedling development in *Arabidopsis* and *Cistus incanus*, a host plant to truﬄes (Splivallo et al., 2007; Hung et al., 2014). Nevertheless lower concentrations of 1-octen-3-ol was shown to induce plant defense genes in *Arabidopsis* (Kishimoto et al., 2007). These volatile signals might therefore modulate the host–plant fitness, however, how effective this modulation might be in nature remains to be investigated.

Another group of potential signaling molecules are nitrogen-containing volatiles. Interestingly, these volatiles seem essentially produced by bacteria but not by fungi. In relation to their habitat, rhizosphere bacteria were the best producers of these volatiles (**Figure 5**). Since these bacteria include the Rhizobium genus, members of which are able to fix atmospheric nitrogen and hence literally serve as natural fertilizers for legumes when colonizing their roots (Gage, 2004), it can be speculated that nitrogen-containing volatiles are involved in signaling between Rhizobium and legumes. As in the case of dimethyl disulfide (Meldau et al., 2013), nitrogen-containing volatiles might be directly assimilated by legumes for nutritional purposes, however, they might serve other purposes as well. Demonstrating their exact role as signaling agents will first require deciphering their biosynthesis.

The examples above illustrate how specific or unspecific belowground volatile signals might be. The various ecological roles highlighted here and, in some cases, the ability of different organisms to emit the same signals, suggest the existence of complex volatile-based interaction networks. Demonstrating their specificity will require characterizing full networks of interacting organisms but also concentrations-activity ratios as well as the persistence of volatile signals in soil.

### Could Root Volatiles be Perceived by Neighboring Plants?

Plants are able to communicate belowground with their neighbors through some unknown signals (Dudley and File, 2007; Bhatt et al., 2011; Fang et al., 2013; Schmid et al., 2013b). Genetic relatedness has recently emerged as an important factor governing belowground root–root interactions. For example roots of rice plants belonging to the same genotype were show to grow toward each other whereas those of different genotypes seem to avoid each other (Fang et al., 2013). Another study involving *Cakile edentula* plants illustrated that plant root allocation is influenced by kinship; indeed the authors observed lower root allocation in kin pairs than stranger pairs (Bhatt et al., 2011). The nature of the signals involved in root–root communication has not yet been fully identified, however, root exudates have been recently suggested as possible candidates (Semchenko et al., 2014). Because volatiles can essentially diffuse further in the soil than root exudates, they might also act as signaling agents in root–root communication. We explored this possibility relying here on indirect evidence. Indeed volatile emission patterns of aboveground plant organs were recently shown to be dependent on kinship, with volatile profiles of kins being more similar to each other than those of plants without kinship (but of the same species) (Karban et al., 2014). Our data demonstrates that how plants respond in terms of root biomass/structure to the presence of a neighboring plant is actually influenced to a certain extent by kinship and genetic relatedness (**Figure 6**). Taken as a whole this suggests that volatile signals might indeed be involved in belowground root–root communications. Demonstrating their exact involvement will require profiling root volatiles as a function of genetic relatedness, identifying the signaling agents, and demonstrating their activity.

## Conclusion

The past decade has seen an increasing interest in belowground volatile-based communication among organisms (Kai et al., 2009; Wenke et al., 2010, 2012; Bailly and Weisskopf, 2012; Piechulla and Degenhardt, 2014). Because of the high heterogeneity and large organismic diversity present in the soil, and the potentially humongous diversity of belowground volatiles, it is essential to apply a holistic approach to understand diversity. Such an attempt has been made here essentially relying on the recently published mVOC database of microbial volatiles (Lemfack et al., 2014) and on a limited number of papers describing plant root volatiles. Although our analysis highlights interesting patterns in belowground volatile diversity and distribution, it also cries out for more data. Essentially we might be looking at the tip of the iceberg and estimating total belowground volatile diversity will require characterizing both the emitters and their full volatile spectra. This will be a major challenge considering the huge number of undescribed soil microbes.

## Author Contributions

DS and RS wrote the manuscript with input from the other co-authors. ML and BP acquired the data. DS and RS further analyzed and categorized the data and DS created the illustrations. All authors approved the final version of the manuscript.

## Conflict of Interest Statement

The authors declare that the research was conducted in the absence of any commercial or financial relationships that could be construed as a potential conflict of interest.
